# Physiological Roles of the Rapidly Activated Delayed Rectifier K^+^ Current in Adult Mouse Heart Primary Pacemaker Activity

**DOI:** 10.3390/ijms22094761

**Published:** 2021-04-30

**Authors:** Wei Hu, Robert B. Clark, Wayne R. Giles, Erwin Shibata, Henggui Zhang

**Affiliations:** 1 Biological Physics Group, Department of Physics and Astronomy, The University of Manchester, Manchester M13 9PL, UK; wei.hu-3@postgrad.manchester.ac.uk; 2 Department of Physiology and Pharmacology, Cumming School of Medicine, University of Calgary, Calgary, AB T2N 4N1, Canada; rclar@ucalgary.ca (R.B.C.); wgiles@ucalgary.ca (W.R.G.); 3 Department of Physiology, Carver School of Medicine, University of Iowa, Iowa City, IA 52242, USA; erwin-shibata@uiowa.edu; 4 Key Laboratory of Medical Electrophysiology of Ministry of Education and Medical Electrophysiological Key Laboratory of Sichuan Province, Institute of Cardiovascular Research, Southwest Medical University, Luzhou 646000, China

**Keywords:** mouse heart, sino-atrial node, SAN, mathematical modeling, spontaneous pacemaker activity, repolarization, K^+^ currents, rapid delayed rectifier, I_Kr_

## Abstract

Robust, spontaneous pacemaker activity originating in the sinoatrial node (SAN) of the heart is essential for cardiovascular function. Anatomical, electrophysiological, and molecular methods as well as mathematical modeling approaches have quite thoroughly characterized the transmembrane fluxes of Na^+^, K^+^ and Ca^2+^ that produce SAN action potentials (AP) and ‘pacemaker depolarizations’ in a number of different in vitro adult mammalian heart preparations. Possible ionic mechanisms that are responsible for SAN primary pacemaker activity are described in terms of: (i) a Ca^2+^-regulated mechanism based on a requirement for phasic release of Ca^2+^ from intracellular stores and activation of an inward current-mediated by Na^+^/Ca^2+^ exchange; (ii) time- and voltage-dependent activation of Na^+^ or Ca^2+^ currents, as well as a cyclic nucleotide-activated current, I_f_; and/or (iii) a combination of (i) and (ii). Electrophysiological studies of single spontaneously active SAN myocytes in both adult mouse and rabbit hearts consistently reveal significant expression of a rapidly activating time- and voltage-dependent K^+^ current, often denoted I_Kr_, that is selectively expressed in the leading or primary pacemaker region of the adult mouse SAN. The main goal of the present study was to examine by combined experimental and simulation approaches the functional or physiological roles of this K^+^ current in the pacemaker activity. Our patch clamp data of mouse SAN myocytes on the effects of a pharmacological blocker, E4031, revealed that a rapidly activating K^+^ current is essential for action potential (AP) repolarization, and its deactivation during the pacemaker potential contributes a small but significant component to the pacemaker depolarization. Mathematical simulations using a murine SAN AP model confirm that well known biophysical properties of a delayed rectifier K^+^ current can contribute to its role in generating spontaneous myogenic activity.

## 1. Introduction

Electrophysiological investigation of the mechanisms that are responsible for myogenic beating, or intrinsic spontaneous pacemaker activity, in vertebrate hearts started with the first successful microelectrode recordings from the leading or primary pacemaker region located high in the right atrium. Intracellular recordings from the amphibian sinus venosus [1], and independent studies of the rabbit sinoatrial node [2] both clearly revealed a slow, progressive depolarization during the diastolic period and showed that this pacemaker potential led up to a distinct threshold for firing regenerative action potentials. Similar, now classical studies also revealed reversible changes in the pacemaker depolarization and action potential caused by transient activation of the autonomic nervous system, for review see [3,4]. 

Our main goal was to evaluate the roles of a time- and voltage-dependent K^+^ current denoted I_Kr_ and often referred to as hERG or mERG [5] in regulating the electrophysiological activity in the adult mouse sinoatrial node (SAN).

### 1.1. Background Information

To provide essential perspectives for this study, we begin with a summary of key advances in experimental work and related mathematical modeling that has revealed essential aspects of the ionic basis for spontaneous pacing in the SAN. The late Professor H. Irisawa led a multidisciplinary group that made a number of seminal discoveries concerning the changes in transmembrane ionic currents that are responsible for the pacemaker depolarization and action potential in the rabbit SAN [4]. Early papers from this laboratory clearly defined the most important characteristics of primary or leading spontaneous pacemaker activity, and also revealed differences between this electrophysiological profile and secondary or follower pacemaking [6]. In addition, the Irisawa group successfully applied the voltage clamp method to small strips of spontaneously active tissue from the adult rabbit SAN. They were also among the first to isolate single pacemaker cells and apply patch clamp methods [4]. Their papers identified essential Ca^2+^ and K^+^ currents in these spontaneous myocytes and demonstrated how interactions between these and other transmembrane currents were functionally important. Key aspects of their original descriptive microelectrode-based data and a comprehensive account of the main changes in ionic currents that are responsible for the pacemaker depolarization and action potential (AP) in the rabbit SAN are the basis of a review by Irisawa and his colleagues [4].

In brief, the available data approximately 25 years ago [7,8,9,10,11,12], was integrated and interpreted mainly in terms of the regenerative upstroke and the plateau of the AP in spontaneously active SAN myocytes being mainly due to the opening and then relatively slow inactivation of L-type Ca^2+^ channels. The repolarization phase of the AP was accounted for by a time- and voltage-dependent activation during the AP plateau of one or more types of ‘delayed rectifier’ K^+^ currents. In addition, the relatively depolarized value of the maximum diastolic potential (MDP), approximately −65 or −70 mV, was accounted for by a background or time-independent Na^+^ current [8]. This current served to hold the MDP significantly depolarized to the K^+^ electrochemical equilibrium potential (approx. −90 mV). 

Importantly, however, a third and seemingly quite unconventional current change in SAN preparations was discovered by the Irisawa group and also by Investigators at Oxford University led by Dr. D. DiFrancesco [4,13,14,15]. This current, denoted I_f_, by Irisawa and his colleagues [4] was shown to be activated by membrane hyperpolarization, and was also modulated (or gated) by changes in intracellular cyclic nucleotide (e.g., cyclic AMP) levels cf [14,16]. Detailed experimental work from groups led by DiFrancesco, Boyett and others has provided strong evidence for the expression of this current in mammalian SAN tissue, and has also revealed that I_f_ is one of the targets of the transmitters released by both branches of the autonomic nervous system [15]. Subsequent experimental work has shown that, I_f_ changes its biophysical properties during early development in ways that add credence to there being an important role for I_f_ in the ontogeny of mammalian cardiac pacemaker activity [17,18]. Recently, the Boyett group has reported that the significant bradycardia which develops as a consequence of required training programs for endurance athletics is accompanied by pronounced but fully reversible down regulation of I_f_ [19,20]. In addition, convincing evidence for a role for I_f_ in primary pacemaker activity has emerged from studies of the significant slowing of heart rate that occurs in conjunction with healthy aging. Specifically, progressive aging is accompanied by both reduced expression of I_f_ and a decrease in the responsiveness of this current to influences of the sympathetic branch of the autonomic nervous system [21,22,23].

### 1.2. Contemporary Hypotheses for Primary Pacemaker Mechanisms

Significant additional insights into the identity and properties of the interacting ionic current changes which can produce the small net inward current that drives the pacemaker depolarization were obtained from detailed studies of a number of different effects of alterations in both extra- and intracellular Ca^2+^. These important findings can be summarized in terms of: (i) effects of changes in intracellular Ca^2+^ [Ca^2+^]_i_; (ii) discovery of distinct contributions of Ca^2+^ fluxes through two different types of Ca^2+^ channels (L-type and T-type) and (iii) recognition of direct and indirect effects on the AP waveform and pacemaker depolarization of Ca^2+^ release from intracellular stores. Initially, the Irisawa group [4,24] and Satoh [12] reported that changes in [Ca^2+^]_i_ in rabbit SAN pacemaker myocytes could significantly alter the voltage dependence of I_f_, and the delayed rectifier K^+^ currents, as well as modifying the inward current generated by the Na^+^/Ca^2+^ exchanger. Either acting alone, or in combination, these effects of [Ca^2+^]_i_ were shown to significantly modulate the pacemaker depolarization [4]. 

These investigators were also the first to clearly demonstrate separate, and additive, functional roles of transmembrane Ca^2+^ influx through both conventional or L-type Ca^2+^ channels, and T-type Ca^2+^ channels [12,25]. During each pacemaker cycle, this increased Ca^2+^ influx steepened the rate of the diastolic or pacemaker depolarization, altered the firing threshold for the action potential and also resulted in a faster rate of the initial depolarization phase of the action potential, for review see [4]. Key aspects of these Ca^2+^-mediated contributions to the overall mechanism for myogenic spontaneous pacing were confirmed and placed in the context of Ca^2+^ channel Molecular Biology by the work of Chiamvimonvat et al. [26]; as well as Mangoni, Nargeot and their colleagues [27,28,29]. These insights were made possible by selective genetic manipulation of the expression of L- and T-type Ca^2+^ channels in the adult mouse SAN. Additional evidence for the requirement of Ca^2+^ influx through L-type Ca^2+^ channels in SAN function had been obtained previously by a number of investigators studying both the amphibian sinus venosus [9,10] and the mammalian SAN in response to sympathetic nerve stimulation or application of isoproterenol, for review see [30,31]. Activation of the sympathetic nervous system consistently resulted in a dramatic (2- to 4-fold) augmentation of the L-type Ca^2+^ current. This effector of autonomic (adrenergic) stimulation significantly promoted spontaneous pacing as well as increasing the height and plateau of the action potential [7,30,31]. 

A third way in which changes in Ca^2+^ is known to be able to contribute to normal and abnormal cardiac automaticity is by altering Ca^2+^ release from intracellular stores, mainly from the sarcoplasmic reticulum (SR). This somewhat complex mechanism was first identified as a causative factor in abnormal supraventricular ‘automaticity’ by the Blatter group [32] based on their analyses of ‘latent’ pacemaker activity in the cat atrium. Lakatta and his colleagues first reported evidence for this type of mechanism in SAN tissue. Their extensive work [33,34,35] has identified many (if not all) of the functional components of a so-called ‘Ca^2+^ clock’ mechanism that can augment or initiate spontaneous pacing in the SAN. Under most circumstances, this Ca^2+^ clock mechanism appears to be functionally separate from the ‘membrane clock’ or interacting transmembrane current changes produced by Ca^2+^, K^+^ and I_f_ channels that are also essential for primary pacemaker activity [36]. 

Depending upon the experimental SAN preparation being studied, Ca^2+^-induced Ca^2+^ release from the SR can either significantly increase the Na^+^/Ca^2+^ exchange current or activate separate nonspecific cation-selective channels that produce transient inward currents at membrane voltages in the range of the pacemaker depolarization [37]. The recognition of involvement of Ca^2+^ release from the SR, coupled with the ability of mammalian pacemaker myocytes to signal changes in Ca^2+^ levels within the SR to the surface membrane, resulted in a search for so-called ‘store operated Ca^2+^ channels’ in mouse SAN myocytes. Interesting results published by Ju et al. [38] provided clear evidence for store operated Ca^2+^ influx in mouse SAN and suggested that this was mediated by channels in the TRP family, likely to be the TRPC variant. 

### 1.3. Na^+^ Channels Can Support SAN Excitability

In both mouse and rabbit SAN myocytes, Na^+^ channels are expressed and Na^+^ currents can contribute significantly to the spontaneous activity profile, mainly by altering heart rate [39,40]. In fact, both the cardiac and the nerve isoforms of the Na^+^ channel alpha subunit (Na_v_ 1.5 and Na_v_ 1.2, respectively) are expressed. Activation of these conductances in SAN tissue can generate a small but significant transient inward current that enhances excitability [39]. It is also noteworthy that mutations in Na_v_ 1.5 or functional/epigenetic down regulation of this current is one of the known causes of intermittent or slow pacing in the human. This is denoted sick sinus syndrome [41,42]. Finally, in SAN preparations obtained from adult rat and guinea pig hearts, an additional, and perhaps essential, transmembrane current is generated by a steady- or time-independent influx of Na^+^ through channels. These appear to be different from either Na_v_ 1.5 or Na_v_ 1.2 [43,44].

### 1.4. Mathematical Models of SAN Pacing

#### 1.4.1. Net Current Changes

Much of the recent work concerning SAN pacemaker mechanisms under both basal conditions, and in settings that include autonomic nervous system activation have focused on the contributions of the ‘Ca^2+^ clock’ mechanism versus, or in conjunction with, current changes due to I_f_ [45,46]. However, virtually all well documented and fully integrated mathematical models of adult rabbit or mouse SAN pacemaker activity are based on significant Na^+^, Ca^2+^ and K^+^ current changes; as well as contributions from Na^+^/Ca^2+^ exchanger and Na^+^/K^+^ pump currents [47,48,49]. It seems likely, therefore, that quit subtle interactions among 5 to 7 separate currents generate the net current that is the basis of spontaneous pacing and generation of SAN action potentials that have a significant safety factor for conduction. The regenerative SAN AP gives rise to excitation of the right atrium and contribute importantly to stable heart rates. 

Comprehensive reviews and related mathematical models of SAN pacemaker activity mechanisms [50,51,52,53,54] differ somewhat in their areas of emphasis and reliance on certain electrophysiological and biophysical principles, see [55]. However, the consensus view [56] strongly suggests that no single or dual channel/transporter mechanism is sufficient to produce myogenic pacemaker activity. Rather, and as indicated, an interplay of channel, antiporter, and electrogenic pump-mediated currents (with the details often depending upon species), is responsible for SAN pacemaker activity. The details of the levels of current expression and their interactions vary significantly depending upon the exact location within the anatomical SAN (primary vs. follower regions) and the presence or absence of phasic autonomic nervous system activity, that is, basal autonomic tone.

#### 1.4.2. K^+^ Current Changes in SAN Preparations

A rapidly activated delayed rectifier K^+^ current is expressed in all primary pacemaker myocytes within mammalian SAN preparations [4]. Although this type of K^+^ current has been studied in detail in atrial and ventricular preparations [5], there has been less detailed analysis or mathematical modeling of this type of K^+^ current in cardiac pacemaker tissues c.f. [47,48,49,50]. Here, we have attempted to address key aspects of this knowledge gap by updating and improving our published mathematical model of SAN pacing in the adult mouse heart. Specifically, we have attempted to account for key biophysical properties of this K^+^ current. Our additional analyses and model development concerning I_Kr_ have focused on: (i) its rapidly developing inward rectification, (ii) its relatively slow but strongly voltage-dependent deactivation at membrane potentials within the pacemaker range, and (iii) its biexponential kinetics that contribute significantly to action potential repolarization and the initial rate of pacemaker depolarization. Accordingly, we have supplemented published data on I_Kr_ in the adult mouse SAN and incorporated these findings into a previously published mathematical model of spontaneous pacing in the mouse SAN [52] (see below for details). 

## 2. Results

### 2.1. Electrophysiological Studies of Adult Mouse SAN Pacemaker Myocytes

**Figure 1:**
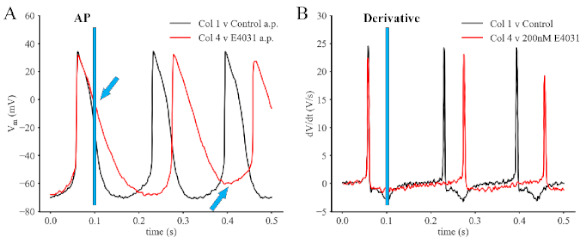
Effects of E4031 on the Action Potential and Pacemaker Depolarization in Adult Mouse Sino Atrial Node (SAN) Primary Pacemaker Myocytes. The traces in Panel (**A**) show three superimposed spontaneous action potentials and pacemaker depolarizations under control conditions (black) and after the application of 200 nM E4031 (red). The vertical blue line denotes the slowing of repolarization and broadening of the action potential duration. The blue arrow at 0 mV marks this effect and the second blue arrow at −60 mV denotes the E4031-induced depolarization of the maximum diastolic potential. E4031 is a quite selective blocker of the HERG K^+^ current. The superimposed traces in Panel (**A**) consist of two sets of superimposed action potentials; and in Panel (**B**) the corresponding first derivative records are shown. Note from the red traces that E4031 markedly reduces the rate of depolarization and repolarization of the action potential.

The experimental component of this study was done to illustrate and confirm the main effects of inhibiting the rapid delayed rectifier K^+^ current, which we denote I_Kr,_ on the spontaneous electrophysiological activity in SAN myocytes. A secondary objective was to reveal key biophysical features of this inwardly rectifying K^+^ conductance that are important for repolarization of the action potential or development of the pacemaker depolarization. As shown in Figure 1, after E4031 (1 μM), a quite selective I_Kr_ blocker, [5,57,58], was added to the normal Tyrodes superfusate, the action potential (AP) lengthened significantly and the maximum diastolic potential (MDP) moved approximately 5–7 mV in the depolarizing direction. Both of these effects on the spontaneously beating SAN electrophysiological profile are designated by the two blue arrows in Figure 1, Panel A. In these records, the control or baseline record is shown in black; and data obtained in E4031 (1 μM) is plotted in red. The corresponding superimposed dV/dt records obtained from the AP (Panel B) highlight the E4031-induced slowing of the rate of AP repolarization. In addition, the decrease in the maximum dV/dt obtained from the upstroke of the APs reveals the expected reduction as a result of the MDP being depolarized from −70 mV to approximately −60 mV.

**Figure 2:**
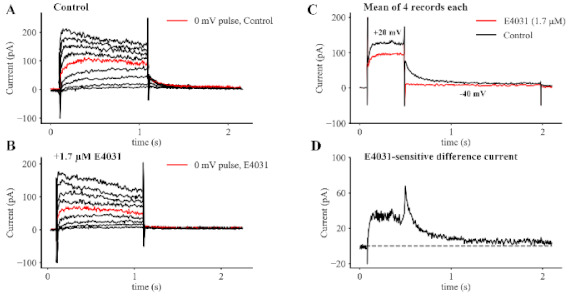
Characterization of the Kinetic Properties of the Rapid Delayed Rectifier K^+^ Current, HERG, in Primary Pacemaker Myocytes from Adult Mouse SAN. The nine superimposed current records in Panel (**A**) show the time- and voltage-dependent activation and deactivation of HERG in response to 1 s rectangular depolarizing volt-age clamp pulses applied in 10 mV increments from a holding potential of −60 mV. The corresponding records in Panel (**B**) were obtained approximately 5 min after the application of the HERG inhibitor, E4031, (1.7 M). In each panel the red traces de-note the currents in response to the depolarization to 0 mV. The records in Panels (**C**) and (**D**) illustrate analysis of these records to identify and characterize the HERG cur-rent as a ‘difference current’ by subtracting records obtained in E4031 from those obtained under control conditions. In Panel C, the two sets of superimposed current records are averages of four traces generated in response to 200 ms depolarizations to +20 mV from a holding potential of −40 mV. The averaged record in red was obtained after application of E4031 and shows significant inhibition of the outward current generated by the depolarization and complete block of the deactivating ‘tail current’ due to HERG. Panel (**D**) shows the E4031-sensitive difference current and depicts two of its major characteristics: inward rectification upon activation, and a prominent, relatively slowly decaying tail current (see Results).

To confirm our previous findings and to identify the main biophysical characteristics of the E4031-sensitive K^+^ current that is responsible for the AP changes shown in Figure 1, single SAN myocytes were studied using standard patch clamp methods. Panels A and B of Figure 2 each consist of a family of control K^+^ current records (Panel A) as well as corresponding current traces recorded after E4031 (1.7 μM) application (Panel B). From comparison of these two data sets, it is apparent that E4031 blocks some, but not all, of the time- and voltage-dependent outward current. In this protocol, each SAN myocyte was held at −60 mV, and progressively larger 1 s depolarizing rectangular voltage command steps were applied to clamp the membrane voltage in the range −40 to +40 mV and then step back to −60 mV. In this Figure, the red traces are included to provide a qualitative impression of the changes induced by E4031 at 0 mV. The records in Panel B demonstrate that the outward current that is activated at 0 mV is reduced by about 30%. Note, however, the corresponding deactivating tail current is completely blocked. Important, in fact, defining biophysical properties of this current, for review see [5] are revealed in Panel C. The two superimposed records clearly illustrate the partial block induced by E4031 during the activation phase of this current, and the complete inhibition when the voltage is returned to the holding potential. This strong inhibition is perhaps the best known biophysical feature of this E4031-sensitive current [5]. It is illustrated in Figure 2, Panel B. This ‘difference current’ was obtained by subtracting the black from the red trace in Panel C, and it clearly shows the inwardly rectifying properties (that is, the deactivation tails are larger than the size of the onset or activation component) of this K^+^ current.

**Figure 3:**
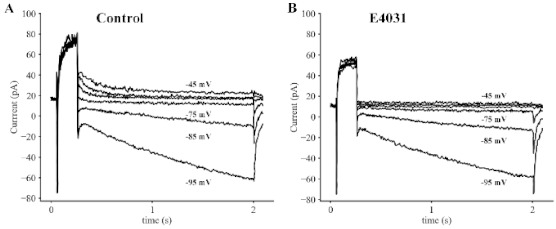
Illustration of the K^+^ Selectivity of HERG in Primary Pacemaker Myocytes from Adult Mouse SAN. The six sets of superimposed traces in both Panels (**A**,**B**) illustrate the deactivation kinetics and reversal potential for the HERG K^+^ current in adult mouse SAN cells. In these paired experiments, 100 ms voltage clamp depolarizations from a holding potential of −60 mV to +5 mV were applied at 0.1 Hz to strongly activate HERG and immediately after each depolarization the membrane potential was returned for 1.9 s to membrane potentials ranging between −45 and −95 mV. Note from the records in Panel (**A**) that the deactivation tail reversed between −75 and −85 mV. The records in Panel (**B**) were generated using an identical protocol to that utilized in Panel A beginning approximately three minutes after the application of 1.7 M E4031. This maneuver was done to separate the deactivation of HERG in the range of membrane potentials −75 to −95 mV from the activation of a separate current, I_f_. (see Results and Discussion).

Results in Figure 1 and Figure 2 confirm and extend our previous studies of K^+^ currents in adult mouse SAN myocytes [57]. Importantly for this study, they also clearly define the changes in the AP and pacemaker depolarization that must be accurately replicated in the mathematical modeling component of this study. Additional characteristics of this current that need to be defined prior to attempting an *in silico* study of its functional roles are: (i) its ion selectivity properties, and (ii) its kinetics in the range of membrane potentials that include those in which the diastolic depolarization develops – approximately −75 to −55 mV. The superimposed families of current records in Figure 3 demonstrate that the deactivating tail currents reverse (from outward to inward) between −75 and −85 mV. This observation confirms findings in our previous study [57], that is, this E4031-sensitive current change is due mainly to a K^+^ conductance. These experimental measurements also show that when membrane potential is hyperpolarized negative to approximately −80 mV, the deactivating inward current tails due to the E4031-sensitive current are superimposed upon a slowly developing *inward current* that grows progressively larger with each stronger hyperpolarization. As expected, our analysis of this current, and the fact that it is consistently identified presence in other primary and secondary cardiac pacemaker tissue strongly suggests that it is the hyperpolarization-activated current, denoted I_f_, as described in the Introduction [4]. Attempts to determine the combination of current changes that result in the net inward current that drives the pacemaker depolarization requires a full understanding of the K^+^ current and the current change due to I_f_ that is illustrated in this Figure.

**Figure 4:**
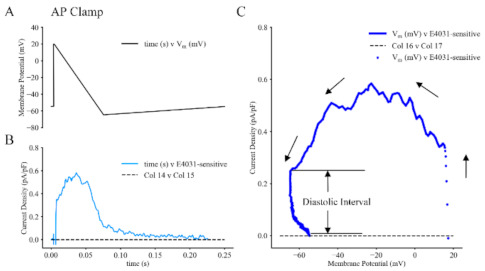
Ramp Voltage Clamp Experiments Done in an Attempt to Reveal the Size and Kinetics of HERG Activation During the ‘Action Potential’ and/or its Deactivation During the Pacemaker Depolarization in Adult Mouse SAN Myocytes. Panel (**A**) shows the ‘AP’ voltage clamp ramp waveform. This was applied at 0.1 Hz from a holding potential of −60 mV. The averaged current records in Panel (**B**) denote the activation and deactivation of HERG during the AP and subsequent slow pacemaker depolarization. The record in Panel (**B**) has been signal averaged and is based on current changes recorded from a total of six different SAN cells. Panel (**C**) is a phase plane plot (dV/dT vs. Vm) that denotes the average (*n* = 10 traces) net E4031-sensitive current during re-polarization of the AP under control conditions (black) and after E4031 application.

As mentioned, our main goal was to assess the functional roles of I_Kr_ in (i) repolarization of the AP and (ii) the subsequent development of the diastolic or pacemaker depolarization in adult mouse SAN myocytes. The relatively small capacitance of these single pacemaker myocytes (25 to 30 pF), and the very slow rate of depolarization (0.1 to 0.5 V/s) that characterizes their pacemaker potential makes it clear that the net current change that is responsible for the pacemaker depolarization will be very small and perhaps even below the resolution of our recording methods. To improve our chances of succeeding with these measurements, a protocol was devised in which the activation and deactivation of I_Kr_ was driven, or regulated by, an artificial voltage clamp command waveform. As shown in Figure 4, it was approximately right triangular in shape, initiated from a stable holding potential of −60 mV and including a rapid depolarization to +20 mV, followed immediately by a linear ramp that repolarized the myocyte to −70 mV. The final component of this ramp clamp protocol consisted of a slow, linear ‘pacemaker depolarization’ back to the holding potential of −60 mV. This command waveform (illustrated in Figure 4A) was applied repetitively at 0.1 Hz and the baseline current records were subtracted from those that remained after E4031 (1.7 μM) addition to the superfusate. A representative difference current is shown in Figure 4B. The combination of these approaches and protocols indeed was able to resolve the approximate size and kinetics of activation, inactivation and deactivation of I_Kr_ within the range of membrane potentials that are relevant to its normal physiological duty cycle and function. In accordance with our previous biophysical analyses [57], the dynamics of these channel-mediated current changes, in relation to the transmembrane voltages are summarized and plotted in Figure 4C. The ordinate denotes changes in current density (pA/pF) and membrane potential (E_m_) on the abscissa. Note that in the range of membrane potential that includes the pacemaker depolarization in the adult mouse heart (denoted ‘diastolic interval’ in this Figure) there is a significant relaxation of outward current.

### 2.2. Mathematical Reconstruction of SAN Action Potential and Pacemaker Depolarization

**Figure 5:**
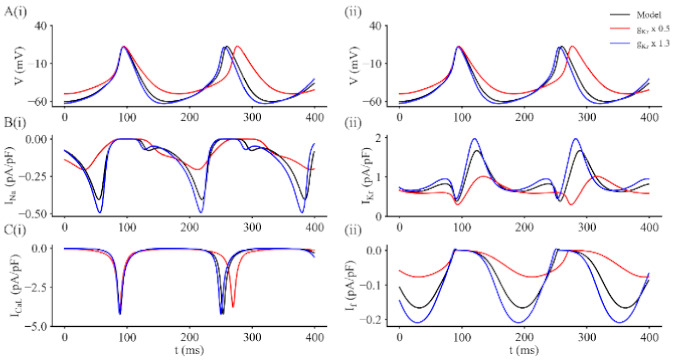
Illustration of simulated action potentials, pacemaker depolarizations and selected underlying ion channel-mediated currents obtained using the updated adult murine SAN myocyte model. In each panel the three superimposed traces represent control or baseline data (black), results obtained after the maximal conductance for I_Kr_ was reduced by 50% (red), and data obtained after I_Kr_ had been increased to 130% of its baseline value (blue). Panels **A**(**i**) and **A**(**ii**) are duplicates; each trace illustrates two action potentials and pacemaker depolarizations. Panel **B**(**i**) shows Na^+^ current, and I_Kr_ records are shown in **B**(**ii**). Panel **C**(**i**) illustrates I_CaL_ traces; and Panel **C**(**ii**) shows changes in the hyperpolarization-activated current, I_f_. Note that when I_kr_ is decreased, the pacemaker rate slows; and the opposite occurs when I_Kr_ is increased. Changes in I_Kr_ also significantly change the maximum diastolic potential (MDP). This effect produces secondary changes in I_Na_, I_CaL_ and I_f_, which in combination, modulates SAN pacemaker activity.

Using this modified mathematical model we have simulated the effects of either blocking or augmenting I_Kr_ on the action potential and pacemaker depolarization in a single adult mouse SAN myocyte. The revised model has also been used to illustrate changes in the major underlying ion channel-mediated currents (I_Na_, I_CaL_) during the spontaneous pacemaker depolarization and action potential. These results are shown in Figure 5. The three superimposed sets of spontaneous action potentials in Panels A and B illustrate output from the modified model under baseline or control conditions (black); and after the maximal conductance of I_Kr_ (g_Kr_) was increased by 30% (blue); or reduced by 50% (red), respectively. The other panels in this Figure show the changes in selected, relatively large transmembrane ionic currents that are essential for successful mathematical reconstruction of SAN pacemaker activity and action potentials. In Figure 5 this information is illustrated as follows: Panel C, I_Na_; Panel D, I_Kr_; Panel E, I_CaL_; and Panel F, I_f_. Note that in distinction to atrial or ventricular myocytes, the sizes or densities of I_Na_ and I_CaL_ are very similar; I_Kr_ and I_f_ are also similar in size, although both are much smaller than either I_Na_ or I_Ca_ (see Discussion). Note also that although *increasing* I_Kr_ has only modest effects on the other currents shown in this Figure, a 50% *reduction* in I_Kr_ produces quite marked changes in I_Na_, I_Kr_ and I_f_. These effects are caused mainly by the maximum diastolic potential (MDP) moving in the depolarizing direction (by approximately 15 mV). This important electrophysiological change is very similar to the experimentally recorded data that describes the E4031 effects in our previous paper [57] and in this study.

**Figure 6:**
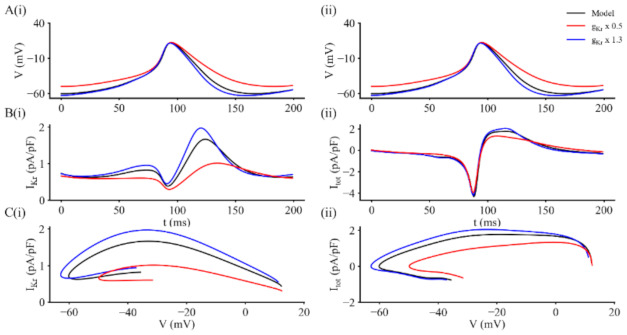
Time course and phase plane plots of I_Kr_ and I_tot_ during a simulated SAN action potential based on our modified mathematical model for this K^+^ current. In these computations, the effects of either a 50% reduction, or a 30% increase in baseline I_Kr_ on the spontaneous membrane action potential (AP) and the net or total ion channel-mediated current (I_tot_) during the AP and pacemaker depolarization were computed. Details of the modifications are in the Methods and the Supplement Section. As in Figure 5, Panels **A**(**i**) and **A**(**ii**) represent identical or duplicate action potential/pacemaker depolarization records presented in this way so that the timing of the ionic current changes shown below can readily be seen. In each panel the three superimposed traces represent control data (black), data obtained from simulations after the maximal conductance for I_Kr_ was decreased by 50% (red) and data sets corresponding to model output after this conductance had been increased to 130% of its baseline value (blue). Panel **B**(**i**) shows I_Kr_ records and in Panel **B**(**ii**) illustrates the net or total transmembrane ionic current (the sum of I_Na_, I_CaL_, I_Kr_ and I_f_). The plots in Panels **C**(**i**) and **C**(**ii**) illustrate our phase plane analyses for I_Kr_ and I_tot_, respectively. Note that the selected decrease in I_Kr_ prolonged APD and depolarized MDP; in combination, these changes reduced the slope of the pacemaker depolarization and slowed heart rate. Increasing I_Kr_ produced opposite effects on APD, MDP, and heart rate.

Based on these data sets, we further considered plausible mechanisms that could be responsible for the effects of alterations in the size of I_Kr_ on the electrophysiological profile of spontaneously active myocytes from the SAN. Some of these results are shown in Figure 6. This Figure consists of one cycle of spontaneous activity of the SAN myocyte, illustrated together with time synchronized records showing the size and time course of changes in I_Kr_; as well as corresponding changes in the total net ion channel-mediated current (I_tot_) during the pacemaker depolarization. To more clearly reveal these changes and their effects, the same primary data is also presented as two phase- plane plots: (i) I_Kr_ vs. E_m_, and (ii) I_tot_ vs. E_m_. 

In Figure 6, Panels A(i) and A(ii) each show the same superimposed pacemaker depolarizations and action potential waveforms under three different conditions: control or baseline (black); following a 30% increase in I_Kr_ (blue); and after I_Kr_ was reduced 50% (red). The corresponding three superimposed I_Kr_ records in Panel B(i) illustrate changes in the size and time course of this current during the action potential and pacemaker depolarization. It is apparent that the 50% reduction in I_Kr_ broadens the action potential and also causes a depolarization of the maximum diastolic potential. Increasing I_Kr_ causes smaller but still detectable changes (increases) in both of these parameters. The phase plane plot in Panel C(i) highlights the changes in I_Kr_ in the membrane potential range −65 to −30 mV that corresponds to the voltage window in which the slow pacemaker depolarization develops. It is apparent that I_Kr_ *increases* during the first two thirds of repolarization (0 mv to −40 mV) and then *decreases* due both to its inward rectification property and its time- and voltage-dependence deactivation. The records in Panels B(ii) and in C(ii) provide analogous information with respect to the behaviour of the net current produced by the modified model ‘in total’. In Panel B(ii) the total current record illustrates the net inward current that produces the action potential upstroke and also shows the time- and voltage-dependent development of the outward current that initiates and then regulates repolarization. Note, also however, that at the 50 ms time point, comparison of the red and blue/black traces reveals that the reduction in I_Kr_ is significant during development of the pacemaker depolarization. This small effect is brought out in a different way (and perhaps more clearly) in the corresponding phase plane plot in Figure 6C(ii).

This initial computational analysis is somewhat revealing, but the alterations in I_Kr_ during the pacemaker depolarization and action potential are complex and biphasic (see Figure S2 in the Supplement Section). The functional roles of I_Kr_ can be illustrated and understood more clearly by: (i) separating the contributions of the fast and slow components of I_Kr_, and also (ii) by varying the total conductance of this rapidly activating, inwardly rectifying K^+^ current. 

**Figure 7:**
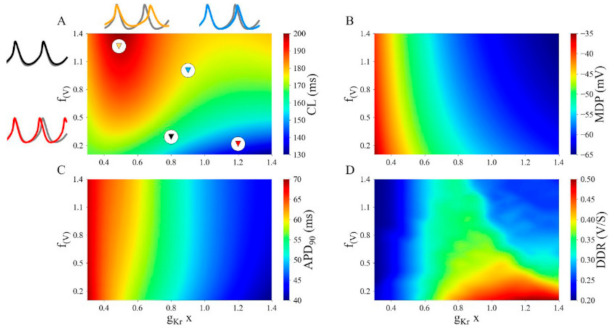
Effects of systematic changes in the maximal conductance for I_Kr_ on SAN electrophysiological parameters. The heat maps in each panel of this Figure depict changes in the parameter named at the right, in response to incremental changes in g_Kr_ in the range 0.3 to 1.4; made in conjunction with changes in the ratio, (f_(v)_), that sets the relative sizes of the slow and fast components of I_Kr_ activation and deactivation. The parameter g_Kr_ was changed from 30% to 140% of its control or baseline values; and f_(v)_ was set to fixed values in the range 0.1 to 1.4. Thus, simulated results were based on relatively small to relatively large ratios of the slow vs. the fast component of I_Kr_. Panel (**A**) denotes changes in cycle length, CL; Panel (**B**) shows alterations in maximum diastolic potential, MDP; Panel (**C**) illustrates changes in APD_90_; Panel (**D**) shows changes in DDR, the slope of the diastolic depolarization.

We have attempted to capture and reveal important dynamic features of the ways in which I_Kr_ can modulate the electrophysiological properties of the SAN using an approach that is based on a ‘population of computations’. In these sets of simulations, f_(V)_, the parameter that defines the relative amounts, or ratio, of the fast and slow components of I_Kr_, was varied between 0.2 and 1.4; and the maximal conductance of I_Kr_, g_Kr_, was scaled by a parameter that was allowed to change between 0.4 and 1.4 of its baseline value (defined as 1.0). These patterns of results are presented in a heat map format in each of the four panels in Figure 7. In this 2-D parameter space, the changes in the overall profile of the electrophysiological activity in the SAN is illustrated in terms of changes in four separate parameters. These are: cycle length (CL) or heart rate (Panel A); maximum diastolic potential (MDP), Panel B; the AP duration at 90% of full repolarization, APD_90_ (Panel C); and rate of pacemaker or diastolic depolarization (DDR), Panel D. In each heat plot the red/orange colours denote an increase or the maximal effect while the blue/green colours denote a minimal change. Beside each panel in this Figure the changes in the parameter of interest is accompanied by a specific colour key. Note that the interaction of the two parameters that regulate I_Kr_, can give rise to nonlinear and, in fact, biphasic modulation of cycle length or heart rate (CL); the rate of the diastolic depolarization (DDR); the action potential overshoot (OS); and the dV/dt_max_ of the action potential upstroke or initial depolarization. In contrast, the interactions between these two parameters appeared to be monotonic when judged in terms of changes in the maximum diastolic potential (MDP); the action potential threshold or takeoff potential (TOP); as well as both APD_50_ and APD_90_. Thus, not only is I_Kr_ an important contributor to AP repolarization, its relatively slow and strongly voltage-dependent deactivation kinetics result in this K^+^ current being an important regulator of the slope of the pacemaker potential or diastolic depolarization (see Discussion).

## 3. Discussion

### 3.1. Summary of Main Findings

The experimental results in Figure 1, Figure 2 and Figure 3 confirm that a rapidly activating delayed rectifier, K^+^ current, I_Kr_, is expressed in myocytes that have characteristics of primary pacemaker cells from the adult mouse SAN; and also provides new information concerning its K^+^ selectivity, inward rectifying properties and sensitivity to the I_Kr_ blocker, E4031. These results, obtained using both rectangular and ramp voltage clamp command waveforms reveal essential roles for I_Kr_ in repolarization of the AP and in strongly modulating the value of the maximum diastolic potential (MDP) in mouse SAN pacemaker myocytes. Data sets from our ramp voltage clamp studies also reveal that deactivation of I_Kr_ is one of the current changes that regulates the slope of the spontaneous pacemaker depolarization. 

Complementary mathematical simulations, which were done using our modified model of the AP and pacemaker depolarization in adult mouse heart SAN myocytes confirm that the I_Kr_ data obtained in our experiments would be expected to produce significant changes in AP duration, the value of the MDP and the slope of the pacemaker depolarization. Based on these simulations when I_Kr_ is either increased or decreased by approximately 50% pacemaker activity changes significantly (Figure 5). This pattern of results reveals that not only the size of but also its slow deactivation kinetics are important regulators of myogenic pacemaking in adult mouse SAN. In summary, our study shows that in primary pacemaker cells from the adult mouse SAN, I_Kr_ qualifies as one of the ‘pacemaker currents’. 

### 3.2. Relationship to Previously Published Work

Previous voltage clamp analyses of the transmembrane ionic current in myocytes from the rabbit SAN clearly established the expression of I_Kr_ in these cells [59]. Boyett and colleagues confirmed this finding, but also reported that the expression level of I_Kr_ was variable when myocytes from distinct anatomical regions of the rabbit SAN were selected for study [58]. Verheijck et al. [60,61] showed that in rabbit SAN preparations, block of I_Kr_ by E4031 not only lengthened the AP duration and resulted in a depolarization of the MDP, but also significantly altered the ability of the SAN to effectively excite or drive the right atrium. This important finding was consistent with previous reports showing that since the atrium had a more negative (or hyperpolarized) resting potential than the MDP in the SAN, electrotonic influences of the atrium could modulate SAN spontaneous activity [62].

Published results that define the molecular identity of the K^+^ channels in tissue from the SAN of adult mouse and rabbit hearts are also relevant to the interpretation of the results from our study. Specifically, Marionneau et al. [63] and Tellez et al. [64] identified similar but not identical I_Kr_ transcripts in mouse and rabbit pacemaker tissue. Tellez et al. [64] also demonstrated that there were important differences in I_Kr_ expression levels between atrial and SAN tissues. These interesting findings were confirmed and extended significantly by the *tour de force* study of Linscheid et al. [56] which utilized quantitative proteomics, coupled with single nucleus transcriptomics and bioinformatics, to fully characterize the spectrum of ion channel, ion exchanger and electrogenic pump transcripts in cardiac pacemaker (mouse SAN) in comparison with atrial tissues. Importantly, in the context of our results, I_Kr_ was one of the K^+^ currents that was identified in SAN. Importantly, activation and deactivation dynamics played a functional role in the mathematical reconstruction [52] of AP repolarization and the pacemaker depolarization in this study.

A third experimental paradigm, and electrophysiological data sets that are directly relevant to the findings of our study are those that have made use of the ‘dynamic action potential clamp’ technique to identify key features of I_Kr_ when it is obtained as an E4031-sensitive ‘difference current’ [65,66]. These findings provided an important precedent for some of our results (Figure 4) by showing that an SAN-like AP applied as a voltage clamp command signal activates I_Kr_ current changes. These records show: (i) rapid activation shortly after the upstroke of the action potential, (ii) a nonlinear outward current that can trigger repolarization; (iii) and decline of this current (both to inactivation and deactivation) during final repolarization and also during the pacemaker depolarization. Other detailed analyses of the features of I_Kr_ [67,68,69] have drawn attention to the importance of identifying the size and kinetics of I_Kr_ at physiological (36 °C–37 °C) as opposed to room temperatures (22 °C–23 °C). Additional studies also revealed that the rapid activation and inwardly rectifying properties of I_Kr_ needed to be taken into account in the design of stimulus patterns and voltage clamp waveforms for valid identification of I_Kr_ properties [70].

### 3.3. The SAN Substrate

Interpretation of our results and the ability to place them in the context of previous studies of SAN pacemaking mechanisms in adult mammalian hearts requires recognition that in both the mouse and the rabbit, as well as in the region of the human right atrium that is the SAN, there is very considerable heterogeneity as judged by differences within this 3-D syncytium in: (i) myocyte size, (ii) types of cells (myocytes vs. fibroblasts) and (iii) proximity or spacing of these various cell types [71,72,73]. With respect to the insights gained from our study, perhaps the most important distinction concerns the differences between the very small region of the anatomical SAN that ‘fires first’; that is, it exhibits leading pacemaker activity. It is well known that a much larger region of SAN tissue also exhibits a distinct diastolic depolarization, but that this ‘secondary or follower region’ always fires its action potentials after the myocytes in the primary pacemaker region [6]. In SAN tissues, such as the ones found in human or canine myocardium, this primary/secondary pacemaker ordering or hierarchy and their interactions have been described as ‘redundant and diverse intranodal pacemakers’ [74]. Both regions are characterized by sparse and spatially specific connexin-mediated intercellular connections compared with other tissues (e.g., atria and ventricles) within mammalian hearts [75]. Finally, we note that the SAN is extensively innervated by the autonomic nervous system, and that both its cholinergic and its sympathetic branches terminate in spatially discrete areas within the SAN. I_Kr_ is modulated by functional linkages to both alpha and beta adrenergic receptors [5,76]. This modulation (and independent regulation of I_Kr_ ion channel targeting to the myocyte surface membrane), can play an important role in the adrenergically mediated and I_Kr_-dependent regulation of AP repolarization and pacemaker rate, perhaps including the adrenergic effects that are associated with circadian rhythm [77]. It is also now recognized that the anatomical SAN includes significant populations of fibroblasts [78,79]. These fibroblasts have the potential to form insulating barriers that alter conduction speed and pathways, as well as to serve as significant current sources through release of paracrine substances in response to the phasic stretch associated with the heart beat [80]. 

### 3.4. Closing Perspectives and Limitations of This Study

As investigators continue to attempt to identify fundamental mechanisms that underlie or modulate the pacemaker depolarization in myocytes from the leading or primary region of the SAN in mammalian hearts, it will continue to be important to be certain that, in fact, only the primary pacemaker phenotype of the SAN myocyte is being studied. In the case of the mouse heart, very detailed descriptions of pacemaker myocyte isolation procedures are available [56,81]. However, the distinction between primary and secondary pacemaker myocytes can be difficult to achieve or to be certain of in mouse SAN, and when pacemaker mechanisms in larger mammalian hearts (rat, rabbit or guinea pig) are being studied. A classical observation by Marshall [82] published in 1957, contains an important insight. In that paper, changes in the electrophysiological phenotype of the rabbit SAN were studied using conventional microelectrodes in the setting of progressively decreased temperature of the superfusate. The results revealed a small region of the rabbit SAN that continued to beat spontaneously at much lower temperatures than the remainder of the anatomical SAN tissue. This criterion has been used to advantage in our studies of primary pacemaking in the amphibian *sinus venosus* (Shibata and Giles, unpublished). 

A remaining challenge is that the individual current changes, or indeed their sum, that are responsible for primary pacemaking in a mammalian single SAN myocyte is very small—perhaps only 5 pA in total. Our work (see Figure 4) has succeeded in resolving the I_Kr_ component of this net current change. It is interesting to note that for their studies of an analogous pacemaker phenomena in the CNS, Khaliq and Bean [83] have developed a useful, improved approach. Their quantitative analysis of the ionic mechanisms that are responsible for the pacemaker depolarization in tegmental neurons (and related simulations) relied upon application of repetitive voltage ramps applied at a range of dV/dt values. They identified a difference current that could convincingly be assigned a novel functional role in this neurogenic pacing. This advance is important, since even very small changes in I_Kr_ can not only modulate SAN pacemaker activity but also offer an important safety factor for the coupling of the SAN to the atria, hence promoting supraventricular conduction [84,85]. 

We acknowledge that our conclusion regarding the functional roles of I_Kr_ in AP repolarization and in regulating the rate of the spontaneous pacemaker depolarization may only apply to the adult mouse SAN. However, this is somewhat unlikely even considering the exceptionally high heart rate in the mouse (approx. 500–600 per minute) and its correspondingly brief AP duration and short inter-beat or diastolic intervals. Although a significant functional role for I_Kr_ may a priori be considered to be minimal, a number of properties of I_Kr_ are well suited for it to be importantly involved. These include: (i) its very rapid activation, even on the time course of the SAN AP; and (ii) its kinetics of deactivation that, in fact, are sufficiently rapid to be able to contribute to the slope and the contour of the pacemaker depolarization. We cannot be certain, however, that the molecular identify of I_Kr_ in the mouse heart is identical with the analogous K^+^ current in rabbit or human SAN. Indeed, the available data (reviewed in [5,86]) strongly suggest that the alpha subunits of the ERG transcripts that are responsible for I_Kr_ differ significantly between mouse (mERG) and human (hERG) hearts. It is also known that the protein complex that generates the I_Kr_ current includes functional beta subunits and that these may also differ across mammalian species. Nonetheless, the significant similarities in the electrophysiological and biophysical properties of I_Kr_ in mammalian SAN suggests that our results need to be seriously considered when seeking to understand or fully explain the ionic basis of the mammalian SAN pacemaker depolarization. Improved functional insights yielded by basic science experiments and clinical investigations will continue to form the basis of contemporary reviews and teaching documents [87] concerning the genesis of spontaneous pacemaker activity and action potentials in mammalian hearts.

## 4. Methods

### 4.1. Mouse SAN Myocyte Isolation

The methods that were used to dissect the sinoatrial node (SAN) tissue, locate the leading or primary pacemaker site, enzymatically isolate the myocytes and then place them in a superfusion chamber have been described in detail in our previous publication [57].

### 4.2. Patch Clamp Recordings and Data Analysis

The electrophysiological methods that were used in this study and the details of the off-line data analysis are as described in our published paper [57]. All of the recordings on which Figure 1, Figure 2, Figure 3 and Figure 4 were based were made four to seven hours after the enzymatic isolation of single myocytes from the SAN tissue was completed. 

### 4.3. Simulation Methods

#### 4.3.1. Parent SAN Myocyte Model

The mathematical model originally developed by Kharche et al. [52] for simulation of the action potential and pacemaker depolarization in myocytes from the adult mouse SAN was used as the starting point for this study. Our main goal was to investigate in detail the roles of the rapidly activating delayed rectifier K^+^ current, HERG or I_Kr_, in AP repolarization and the spontaneous pacemaker depolarization in the adult mouse heart. The Kharche et al. model [52] was chosen as a starting point based on its successful peer review and publication, and its extensive validation against key features of experimental data describing most of the major transmembrane currents in mouse SAN myocytes. A detailed description and validation of this model has been published [52]. We recognized, however, that this model needed to be updated and modified so that it could adequately account for essential biophysical characteristics of the mammalian heart, HERG K^+^ current.

#### 4.3.2. Revision and Update of the Mathematical Descriptors for I_Kr_ or HERG in Mouse SAN

Our original model for the mouse SAN myocyte included only a quite simplistic mathematical description of the kinetics and ion transfer relationship for HERG or I_Kr_. We now recognize that the kinetics of I_Kr_ are complex. Both its onset and decay kinetics are best described by an expression consisting of the sum of two exponential processes. This was described in our previous paper Clark et al. [57] but was not explored in detail. Accordingly, the mathematical model developed for use in the present study includes new parameters derived by fitting the model equations to additional experimental data for I_Kr_ (see Figure S1A,B in the Supplement Section). This fitting procedure and optimization method has been published by Luersen et al. [88].

In the updated I_Kr_ model, we assume two kinetically distinct components of I_Kr_ with the same steady-state voltage dependence (p_a,f∞_, p_a,s∞_) for their fast and slow activation variables (p_a,f_, p_a,s_). Importantly, however, their voltage-dependent time constants, τ_p,af_ and τ_p,as_, respectively differ substantially. In the revised model the fractional ratio between the slow and fast components of K^+^ fluxes that generate the macroscopic I_Kr_ is designated as f_v_. The parameters for τ_p,af_ and τ_p,as_ equations were derived by fitting the model equations to our previously published experimental data in Clark et al. [57]. An example of these current records and the fitted relationships is shown in Figure S1C. The voltage-dependence f_v_ was also derived from the experimental data of Clark et al. [57]. In our simulations that were done to investigate the role of f_v_ in modulating SAN action potentials, and pacemaker depolarizations, the values of f_v_ were selected in the range 0.1–1.4. During this model development and validation, the same voltage clamp protocol as used as was used in the experiments published by Clark et al. [57] was implemented to simulate I_Kr_ records (Figure S1A). These records were also used to obtain the I–V relationships for I_Kr_. Measurements were made at the end of the test pulse (Figure S1B) for the isochronal I–V relationships; and at the peak of each tail current (Figure S1D) to generate activation curve data. Care was taken to ensure that the simulated results closely matched the experimental data of Clark et al. [57].

The full equations used to generate I_Kr_ are listed below. Details concerning definition and units of the model parameters can be found in Kharche et al. [52].

(1) $$I_{Kr}=g_{Kr}\left(\left(1-f_{V}\right)\times p_{a,f}+f_{V}\times p_{a,s}\right)\times p_{i}\times \left(V-E_{K}\right)$$

(2) $$p_{a,f\infty }=p_{a,s\infty }=\frac{1}{1+e^{-\left(V+21.17\right)/9.76}}$$

(3) $$p_{i,\infty }=\frac{1}{1+e^{\left(V+16.76\right)/19.0}}$$

(4) $$\tau_{pa,f}=\frac{13.63}{0.13e^{V/9.49}+7.3\times 10^{-3}e^{-V/14.84}}$$

(5) $$\tau_{pa,s}=\frac{3.97\times 10^{2}}{5.11e^{V/11.36}+8.25\times 10^{-2}e^{-V/23.71}}$$

(6) $$\tau_{pi}=0.2+\frac{0.9}{0.1e^{V/54.65}+0.66e^{V/106.16}}$$

(7) $$\frac{dp_{a,f}}{dt}=\frac{p_{a,f\infty }-p_{a,f}}{\tau_{p_{a,f}}}$$

(8) $$\frac{dp_{a,s}}{dt}=\frac{p_{a,s\infty }-p_{a,s}}{\tau_{p_{a,s}}}$$

(9) $$f_{v}=\frac{\varphi_{v}}{1+\varphi_{v}}$$

(10) $$\varphi_{v}=\frac{6.24}{57.62e^{V/14.47}+4.8e^{-V/50.32}}$$

#### 4.3.3. Numerical Procedures and Schemes

The equations that compromise the updated model were solved using a fixed time step of 1 × 10^−3^ ms. This was sufficiently small to ensure stable numerical solutions. Each simulation epoch consisted of computing a 20-s train of APs and pacemaker depolarizations together with continuous time records of the selected underlying ion channel-mediated currents. The last 1 s section of this data was selected for presentation and analysis in each Figure.

## Data Availability

Simulation code of the murine SAN cell model can be obtained by sending a request to Professor Henggui Zhang (henggui.zhang@manchester.ac.uk). For the experimental data, Dr. W. Giles has experimental methods and related digital files that provide all available details of the results presented in Figure 1, Figure 2, Figure 3 and Figure 4 of the manuscript.

## Supplementary Materials

The following are available online at https://www.mdpi.com/article/10.3390/ijms22094761/s1.
