# Supplementary material for: Physiological Roles of the Rapidly Activated Delayed Rectifier K+ Current in Adult Mouse Heart Primary Pacemaker Activity
Source: Int J Mol Sci. 2021 Apr 30;22(9):4761. doi: 10.3390/ijms22094761 (PMC8124469; doi:10.3390/ijms22094761)
Supplement: Supplementary file 1 [file ijms-22-04761-s001.zip › ijms-1205238-supplementary.pdf]

Online Supplement

Physiological Roles of the K^+ Current, HERG, in Adult Mouse Heart Primary Pacemaker Activity

Wei Hu [1], Robert B. Clark [2], Wayne R. Giles [2], Erwin Shibata [3] and Henggui Zhang [1,4]

[1] Biological Physics Group, Department of Physics and Astronomy, The University of Manchester, Manchester M13 9PL, UK
[2] Department of Physiology and Pharmacology, Cumming School of Medicine, University of Calgary, Calgary, AB T2N 4N1, Canada
[3] Department of Physiology, Carver School of Medicine, University of Iowa, Iowa City, IA 52242, USA
[4] Key Laboratory of Medical Electrophysiology of Ministry of Education and Medical Electrophysiological Key Laboratory of Sichuan Province, Institute of Cardiovascular Research, Southwest Medical University, Luzhou 646000, China

1. Updated I_{Kr} equation and validation

To update the equations for the rapidly activating delayed rectifier K^+ current, I_{Kr}, we modified the I_{Kr} formulations in our previously published Kharche et al. model [1] for mouse SAN myocyte action potentials. In the updated I_{Kr} model, the parameters for the steady-state voltage dependence of fast and slow activation variables ($p_{a,f\infty}$, $p_{a,s\infty}$), voltage-dependent time constants, $\tau_{p,af}$ and $\tau_{p,as}$, the fractional ratio between the slow and fast components of K^+ fluxes that generate the macroscopic I_{Kr} (f_v), were derived by fitting the model equations to our previously published experimental data in Clark et al. [2]. During the model development and validation, the same voltage clamp protocol as used as was used in the experiments published by Clark et al. [2] was implemented to simulate I_{Kr} records (Figure S1A). These records were also used to obtain the I-V relationships for I_{Kr}. Measurements were made at the end of the test pulse (Figure S1B) for the isochronal I-V relationships; and at the peak of each tail current (panel D of Figure S1) to generate activation curve data.

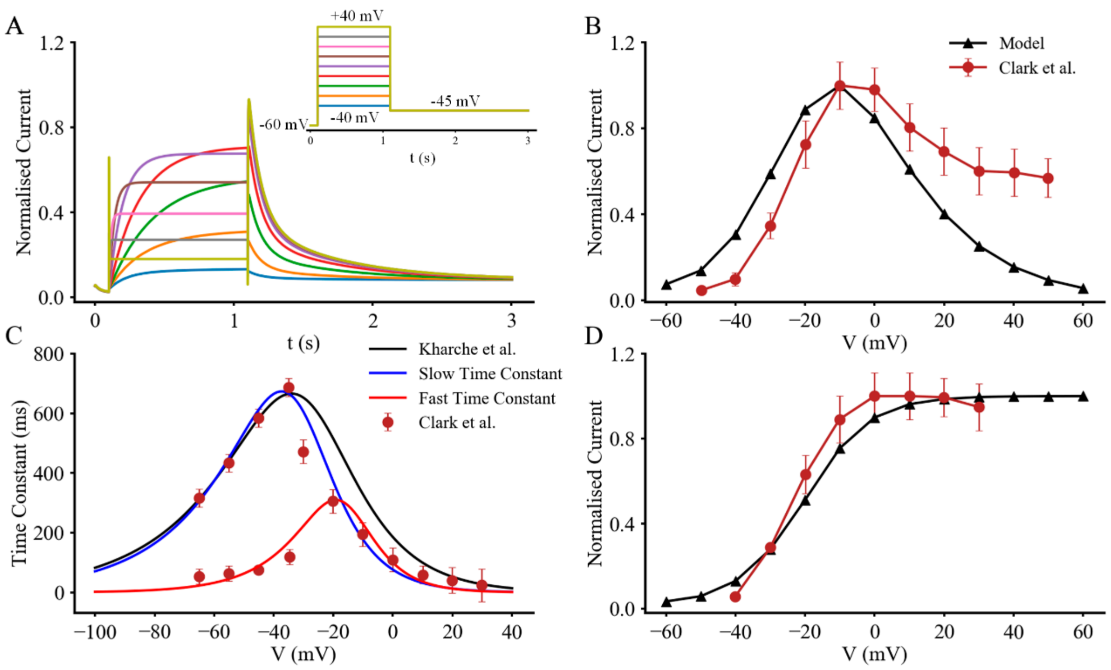

Figure S1. Development and validation of the updated I_{Kr} model.
(A) Simulated time traces of I_{Kr} during the voltage-clamp protocol as shown in the inset. (B) Simulated I-V relationship obtained at the end of testing potentials (black) are compared to experimental data of Clark et al. [2]. (C) Comparison between simulated slow (blue) and fast (red) time constants of the activation variable of the I_{Kr} model and experimental data of Clark et al. [2] (solid circles with error bars). The time constant of activation variable in original Kharche et al. [1] model is also shown for comparison (black). (D) Simulated activation curve obtained at the tail current (black) are compared to experimental data of Clark et al. [2].

Successful incorporation of the updated I_{Kr} formulations into the parent Kharche et al. model, required some additional relatively small changes in other model parameters. The L-type Ca^{2+} channel maximal conductance was reduced scale by a factor of 0.5; the T-type Ca^{2+} channel conductance was increased by a factor of 1.1; and the maximal electrogenic current generated by the Na^{+}-K^{+} pump was scaled by a factor 0.74. These changes were made to make it possible for the characteristics or biomarkers of the simulated pacemaker action potentials (e.g. pacemaking cycle length, maximal diastolic potential, AP overshoot and AP maximal upstroke velocity) generated by the updated model to closely approximate those from the original Kharche et al. model.

2. Modulation of pacemaker action potentials by I_{Kr}

In these simulations, the parameter g_{kr} was modified or scaled in a range including: a 60% reduction, i.e., (g_{Kr} x0.4) to a 40% increase i.e., (g_{Kr} x1.4) as indicated by the labels on the abscissa on each panel in Figure S2. Note that changes in g_{Kr} in the range of (0.5 – 1.4), resulted in corresponding, monotonic increases in cycle length (CL) as shown in panel Ai of Figure S2. These changes were accompanied by a depolarized MDP (panel Aii in Figure S2); an increased threshold for firing or take off potential (TOP) (panel Bi in Figure S2); a reduced rate of the spontaneous pacemaker depolarization (DDR) (panel Bii in Figure S2); and increased AP durations measured at both APD_{90} (panel Ci in Figure S2) and APD_{50} (panel Cii in Figure S2). There was little if any change in the maximum depolarization or overshoot (OS) of the AP as shown in panel Di of Figure S2. However, a small bi-phasic change in the dV/dt_{max} was observed (panel Dii in Figure S2). In contrast, when g_{Kr} was reduced by more than 50%, the computed CL decreased progressively; in part, this was because the MDP moved in the depolarizing direction and thus was closer to the threshold for AP firing that is strongly dependent on two transient inward currents: I_{Na} and I_{CaL}.

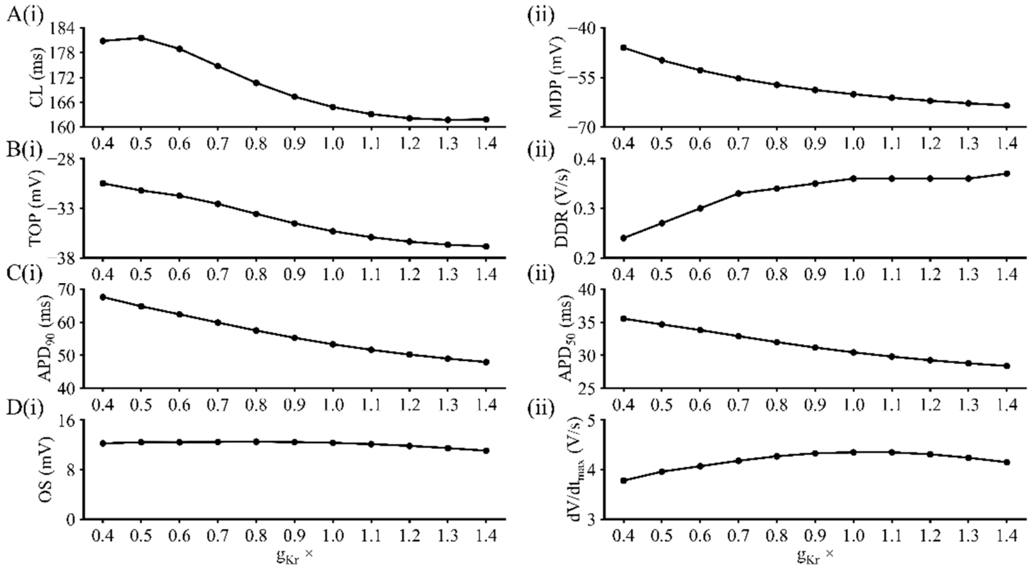

Figure S2. Summary of the effects of systematic scaling of the maximal conductance of I_{Kr}, (g_{Kr}) on action potential descriptors

Effect of a systematic scaling of the maximal channel conductance of I_{Kr} (g_{Kr}) from a 60% reduction (g_{Kr} x0.4) to 40% augmentation (g_{Kr} x1.4) on the characteristics of the pacemaking action potentials. In the scaling range of (0.5 – 1.4), a decrease of g_{Kr} caused a monotonic increase of the CL, which is accompanied by an elevated MDP, increased TOP, reduced DDR, increased APD_{90} and APD_{50}, but unnoticed change in the OS, and a bi-phasic change in the dV/dt_{max}. When g_{Kr} is reduced more than 50% (i.e., x <0.5), the computed CL starts to decrease as the MDP is getting closer to the TOP. (Ai) CL; (Aii): MDP; (Bi) TOP; (Bii) DDR; (Ci) APD_{90}; (Cii) APD_{50}; (Di) OS; and (Dii) dV/dt_{max}.

References

1. Kharche, S.; Yu, J.; Lei, M.; Zhang, H., A mathematical model of action potentials of mouse sinoatrial node cells with molecular bases. American Journal of Physiology-Heart and circulatory physiology 2011, 301, (3), H945-H963.

2. Clark, R. B.; Mangoni, M. E.; Lueger, A.; Couette, B.; Nargeot, J.; Giles, W. R., A rapidly activating delayed rectifier K+ current regulates pacemaker activity in adult mouse sinoatrial node cells. American Journal of Physiology-Heart and Circulatory Physiology 2004, 286, (5), H1757-H1766.
